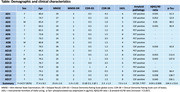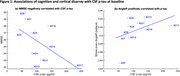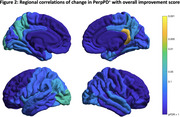# Cortical Microstructure in AD Patients Treated with Lecanemab – Six‐Month Update on an Ongoing Real‐World Observational Study

**DOI:** 10.1002/alz70856_107518

**Published:** 2026-01-09

**Authors:** Gerard R Ridgway, Takashi Nakajima, Mario Torso, Ian Hardingham, Pegah Khosropanah, Kentaro Ohta, Izumi Aida, Steven A Chance

**Affiliations:** ^1^ Oxford Brain Diagnostics, Oxford, United Kingdom; ^2^ National Hospital Organization Niigata National Hospital, Niigata, Japan; ^3^ Niigata National Hospital, Kashiwazaki City, Niigata, Japan

## Abstract

**Background:**

Measures of cortical microstructure from diffusion MRI (dMRI) have been shown to relate to amyloid pathology and to neuroinflammation, and can predict subsequent macrostructural atrophy (Torso et al., 2022, PMID:36281682). We have previously shown practicality of these methods using 1.5T MRI in a real‐world hospital setting (Ridgway, Nakajima, et al., 2023, AAIC; jRCT1032210367).

Anti‐amyloid treatments such as Leqembi (Lecanemab) have been shown to reduce rates of clinical deterioration, but are associated with accelerated brain volume loss, which is presumed to be transient, but is not yet fully understood.

We have commenced a study (https://jrct.niph.go.jp/en‐latest‐detail/jRCT1031240123) to explore the utility of dMRI measures to support early diagnosis and to track treatment response. Information on tissue microstructure is expected to add insights regarding volumetric atrophy or pseudoatrophy.

**Method:**

Patients are recruited at National Hospital Organization Niigata National Hospital, with inclusion and exclusion criteria based on those of the approved drug Lecanemab. Amyloid positivity is confirmed with either amyloid PET or CSF Aβ42/40 ratio < 0.067.

T1‐weighted anatomical MRI (T1w) and dMRI (32‐directions at b=1000 s/mm^2^) are acquired on a 1.5T Philips Ingenia MRI scanner and processed using FreeSurfer, FSL, and proprietary software to produce minicolumn‐associated microstructural measures (AngleR, ParlPD and PerpPD^+^; McKavanagh et al., 2019, PMID:31355989).

**Result:**

The study has enrolled 17 AD patients at the time of analysis, summarised in Table 1. Patients were scanned at baseline, and after 2, 3 and 6 months. 59 total scans were analysed, including 13 6‐month scans.

Figure 1 shows baseline associations of cognition (MMSE; lower scores worse) and cortical disarray (AngleR, higher values worse) with *p*‐tau (higher values worse) for cases with CSF.

Figure 2 shows regional associations of change in PerpPD+ with an overall improvement score that averaged MMSE, MMSE‐DR, CDR‐GS, CDR‐SB and IADL.

**Conclusion:**

We have introduced a promising real‐world observational study that could shed light on the microstructural nature of amyloid‐lowering treatment response and the debate about pseudo‐atrophy.

Microstructural imaging measures are sensitive treatment biomarkers that correlate with clinical changes. Future work with a larger dataset will investigate the potential prediction of clinical change using baseline cortical disarray measurements.